# Bidirectional Relationships and Disconnects between NAFLD and Features of the Metabolic Syndrome

**DOI:** 10.3390/ijms17030367

**Published:** 2016-03-11

**Authors:** Patrick Wainwright, Christopher D. Byrne

**Affiliations:** 1 Clinical Biochemistry, University Hospital Southampton, Tremona Road, Southampton SO16 6YD, UK; patrick.wainwright@uhs.nhs.uk; 2 Nutrition and Metabolism, Faculty of Medicine, University of Southampton, Tremona Road, Southampton SO16 6YD, UK; 3 Southampton National Institute for Health Research Biomedical Research Centre, University Hospital Southampton, Tremona Road, Southampton SO16 6YD, UK

**Keywords:** NAFLD, metabolic syndrome, insulin resistance, PNPLA3

## Abstract

Non-alcoholic fatty liver disease (NAFLD) represents a wide spectrum of liver disease from simple steatosis, to steatohepatitis, (both with and without liver fibrosis), cirrhosis and end-stage liver failure. NAFLD also increases the risk of hepatocellular carcinoma (HCC) and both HCC and end stage liver disease may markedly increase risk of liver-related mortality. NAFLD is increasing in prevalence and is presently the second most frequent indication for liver transplantation. As NAFLD is frequently associated with insulin resistance, central obesity, dyslipidaemia, hypertension and hyperglycaemia, NAFLD is often considered the hepatic manifestation of the metabolic syndrome. There is growing evidence that this relationship between NAFLD and metabolic syndrome is bidirectional, in that NAFLD can predispose to metabolic syndrome features, which can in turn exacerbate NAFLD or increase the risk of its development in those without a pre-existing diagnosis. Although the relationship between NAFLD and metabolic syndrome is frequently bidirectional, recently there has been much interest in genotype/phenotype relationships where there is a disconnect between the liver disease and metabolic syndrome features. Such potential examples of genotypes that are associated with a dissociation between liver disease and metabolic syndrome are patatin-like phospholipase domain-containing protein-3 (PNPLA3) (I148M) and transmembrane 6 superfamily member 2 protein (TM6SF2) (E167K) genotypes. This review will explore the bidirectional relationship between metabolic syndrome and NAFLD, and will also discuss recent insights from studies of PNPLA3 and TM6SF2 genotypes that may give insight into how and why metabolic syndrome features and liver disease are linked in NAFLD.

## 1. Introduction

Non-alcoholic fatty liver disease (NAFLD) is a considerable public health concern, and is the commonest cause for chronic liver disease in the developed world [1,2]. Worldwide prevalence of NAFLD is estimated to be in the region of 20% in the general population [3]. NAFLD represents a disease spectrum ranging from hepatic steatosis, to non-alcoholic steatohepatitis, to cirrhosis, end-stage liver failure and hepatocellular carcinoma. The accepted definition of NAFLD is a hepatic triglyceride content of greater than 5.5%, as determined from analysis of the Dallas Heart Study cohort [4]. The metabolic syndrome is a collection of underlying risk factors for cardiovascular disease with an estimated prevalence in the USA of 34% [5].

The relationship between NAFLD, obesity, insulin resistance and type 2 diabetes is a complex one. NAFLD has traditionally been considered to be the hepatic manifestation of the metabolic syndrome, due to the close association between NAFLD and the various component features of the metabolic syndrome such as abdominal obesity, hypertension, elevated fasting plasma glucose, raised serum triglycerides and low high-density lipoprotein cholesterol (HDL-C) concentrations. Many epidemiological studies have demonstrated an association between NAFLD and the metabolic syndrome [6,7,8].

There is now a growing body of evidence supporting the idea that there is a bidirectional relationship between NAFLD and features of the metabolic syndrome, with insulin resistance being the central pathophysiological process common to both conditions. As such there currently exists and “chicken and egg” debate in the literature regarding the temporal relationship between NAFLD and the metabolic syndrome, with no clear consensus about which is considered to generally occur first. A recent study has demonstrated a reciprocal causality between NAFLD and metabolic syndrome in a Chinese population, with metabolic syndrome being found to have a greater effect on incident NAFLD in terms of causality than NAFLD does on incident metabolic syndrome [9].

In addition to this there are recognised situations whereby there is an apparent disconnect between NAFLD and insulin resistance/metabolic syndrome features, and these generally arise as a result of particular genetic polymorphisms such as in the patatin-like phospholipase domain-containing protein-3 (PNPLA3) gene.

This review will attempt to review the available evidence regarding the bidirectional relationship between NAFLD and components of the metabolic syndrome, as well as to explore the potential disconnects that may exist between the two due to genetic variability and inherited metabolic disease.

## 2. Association between NAFLD and Components of the Metabolic Syndrome

There have been various diagnostic criteria available for the diagnosis of metabolic syndrome, and these have changed subtly over recent years. The most commonly used criteria are those published by the International Diabetes Federation in 2009. It should be noted that these most recent criteria advocate using population- and country- specific definitions for abdominal obesity [10]. Table 1 outlines the various diagnostic criteria available.

NAFLD can occur in individuals who are not obese [11,12], however this is more unusual and generally NAFLD is closely related to increased central adiposity. NAFLD is commonly associated with all of the component features of the metabolic syndrome, and nearly two thirds of people with obesity and type 2 diabetes demonstrate hepatic steatosis [13,14]. One study identified hepatic steatosis via ultrasonography in 50% of patients with hyperlipidaemia [15]. NAFLD is also associated with arterial hypertension and cross-sectional studies have demonstrated that approximately 50% of people with essential hypertension also have NAFLD [16,17]. Importantly, in those people with NAFLD the presence of multiple features of the metabolic syndrome is associated with more severe liver disease and a higher likelihood of progression to NASH and cirrhosis [18,19].

## 3. NAFLD as a Risk Factor for and Precursor to the Metabolic Syndrome

There is evidence to suggest that NAFLD, rather than being simply the hepatic manifestation of the metabolic syndrome, may in fact be a necessary first step in its development.

When the link between NAFLD and insulin resistance was initially described by Day et al, it was proposed as part of a “two hit hypothesis” [20]. Here, the “first hit” was increased triglyceride accumulation as a result of insulin resistance and increased delivery of free fatty acids to the liver, followed by a “second hit” of hepatic oxidative stress resulting in increased lipid peroxidation. This was said to then lead inexorably to hepatocyte injury, inflammation and fibrosis, with the potential for progressive liver damage. It has subsequently been suggested that pathogenesis of NAFLD may in fact reflect “multiple parallel hits” which all contribute to an environment of hepatic inflammation with the involvement of cytokines and adipokines from extrahepatic tissues such as the gut and adipose tissue [21].

From a basic science perspective, there is reason to believe that hepatic lipid accumulation could be a cause and a perpetuating factor for the development of insulin resistance. There is currently much interest in fully elucidating the role that protein kinase C-ε (PKC-ε) may play in this relationship. An elegant study conducted by Samuel et al investigated PKC-ε and how it may link NAFLD and insulin resistance [22]. They observed that rats that were fed a 3 day high-fat diet developed marked hepatic steatosis and hepatic insulin resistance as determined by hyperinsulinaemic-euglycaemic clamp studies. Here, PKC-ε was activated but other forms of PKC were not. Crucially, the authors then went on to attenuate the expression of PKC-ε using an anti-sense oligonucelotide directed at PKC-ε and they noted that this protected the rats from steatosis-induced hepatic insulin resistance and also reversed defects that they had observed in insulin receptor signalling function. It should be noted that both hepatic diacylglycerol and triacylglycerol content were not affected by this intervention suggesting that the hepatic lipid accumulation is a prerequisite for insulin resistance. This relationship has also been investigated in humans, in a study of 37 obese non-diabetic individuals awaiting bariatric surgery [23]. Here it was observed that hepatic diacylglycerol content from liver biopsy specimens was the strongest predictor of insulin resistance and accounted for 64% of the variability in insulin sensitivity. Hepatic diacylglycerol content was strongly correlated with activation of PKC-ε. Given this evidence, a model has emerged whereby increases in liver diacylglycerol content result in activation of PKC-ε, translocation of PKC-ε in the cell membrane, inhibition of hepatic insulin signalling and the resulting generation and maintenance of hepatocyte insulin resistance.

More recently there has been interest in the hepatokine, fetuin B. This compound has been shown to be increased in obese rodents [24]. It has also been shown that overnutrition in experimental mice results in hepatic steatosis, and this alters the hepatocyte protein secretion profile leading to increased secretion of fetuin B [25]. The authors of this study went on to further study the effects of fetuin B *in vivo* and observed that injecting recombinant fetuin B intraperitoneally into mice significantly impaired glucose tolerance when compared with controls. In addition to this, silencing fetuin B gene expression using short hairpin RNA was found to increase glucose tolerance. As such, fetuin B provides an example of how hepatic steatosis can be linked to the development of insulin resistance and thus the metabolic syndrome. Other hepatokines such as FGF21 and selenoprotein P are thought to be play a role in the pathophysiology of insulin resistance with action on the liver and other tissues, however it is less clear how they fit into the relationship between hepatic steatosis and the metabolic syndrome.

It is known that most people with NAFLD also have insulin resistance, however most do not exhibit all of the features of the metabolic syndrome [26]. This could indicate that hepatic steatosis is required as a prerequisite for the development of further metabolic disease such as altered glucose and lipid metabolism. There is now a significant body of clinical evidence for NAFLD preceding, and being a strong risk factor for, development of the metabolic syndrome and its various components. A large prospective cohort study looked at 17,920 individuals from a Han Chinese population and followed them up over a 6 years period [27]. These individuals did not have metabolic syndrome at baseline, and the authors identified NAFLD as an independent risk factor for its development with an adjusted hazard ratio of 1.55 (95% confidence intervals 1.39–1.72). This observation of NAFLD as an independent risk factor for the development of the metabolic syndrome has also been made in a variety of other populations such as North American [28], western Australian [29], Korean [30], Japanese [31] and south Indian [32].

A large prospective cohort study of over 22,000 Korean men demonstrated that NAFLD is an independent risk factor for incident arterial hypertension, and that risk increases with severity of NAFLD [33]. This study replicated the findings of an earlier, smaller prospective study which demonstrated that NAFLD was an independent risk factor for the development of prehypertension [34]. Another prospective cohort study examined 1521 people and stratified them on the basis of their fatty liver index score (a surrogate marker of hepatic steatosis) [35]. It was observed that NAFLD, as diagnosed using fatty liver index score, was an independent risk factor for incident arterial hypertension. Finally, a retrospective cohort study of 11,448 individuals without hypertension revealed that the development of incident fatty liver disease over a five years period was associated with increased risk of incident hypertension [36].

A retrospective study of a Korean occupational cohort of 13,218 individuals observed that development of new fatty liver was associated with incident diabetes [37]. There are many prospective studies in the literature that demonstrate that NAFLD, and the surrogate markers with which it is associated, is a key risk factor and precursor for the development of type 2 diabetes [29,38,39,40,41,42,43,44,45,46]. Table 2 summarises the characteristics of these key studies.

Of particular interest is a longitudinal cohort study in which the authors followed up 358 individuals (109 with NAFLD, 249 without NAFLD) over an 11 years period [29]. After excluding those who had type 2 diabetes at baseline, they observed that those with NAFLD were significantly more likely to develop diabetes during the follow up period than those without. Similarly, they observed the same regarding who would go on to develop the metabolic syndrome. Also, a retrospective study of a Korean occupational cohort of 12,853 individuals demonstrated that the clustering of insulin resistance, overweight/obesity and hepatic steatosis markedly increased risk of incident type 2 diabetes [47]. The fully adjusted odds ratio for those with all 3 factors and risk of incident diabetes at 5 years follow-up was 14.13 (95% confidence intervals 8.99–22.21).

In addition to this, a meta-analysis has been performed recently which concluded that the presence of NAFLD doubles an individual’s risk of developing type 2 diabetes in later life [48]. It would seem that there may be subsets of patients with NAFLD that have different levels of risk of type 2 diabetes, with one small study suggesting that the presence of biopsy-proven NASH is a greater risk factor than steatosis alone [41]. This is consistent with the accepted notion that individuals with nonalcoholic steatohepatitis (NASH) will tend to have a greater burden of metabolic disease.

## 4. Metabolic Syndrome as an Initiating or Aggravating Factor for Liver Disease

In addition to the evidence from the literature that NAFLD may predispose individuals to developing or worsening insulin resistance and the metabolic syndrome, there is also growing evidence that insulin resistance may contribute to progressive liver damage.

Of particular interest is the role played by plasminogen activator inhibitor 1 [49]. PAI-1 is a member of the serine protease inhibitor family, and acts as a key mediator in the fibrinolytic system. In tissues with a significant degree of fibrosis, concentrations of PAI-1 are elevated leading to an inhibition of tissue proteolytic activities, a decreased rate of collagen degradation and increased tissue fibrogenesis [49]. Increased PAI-1 levels are associated with obesity, insulin resistance, type 2 diabetes and dyslipidaemia [50,51]. Specifically it has been shown that PAI-1 concentrations measured in subcutaneous adipose tissue biopsy samples from individuals with nascent metabolic syndrome are significantly higher than those in control samples [52]. It has also been observed in a human hepatocyte cell line that tumour necrosis factor α (TNF-α) is able to induce the expression of PAI-1, leading to increased hepatic fibrosis and atherosclerosis in insulin-resistant individuals [53]. There is also a wealth of evidence in the literature regarding the role of PAI-1 in initiating and perpetuating hepatic fibrosis [49]. As such this provides evidence of a causative role for insulin resistance and obesity in the generation of ongoing hepatic fibrosis.

In addition to this, there is evidence that other inflammatory cytokines originating from white adipose tissue as a result of obesity and insulin resistance may play a significant role in hepatic fibrosis and inflammation. It has been known for some time that white adipose tissue is not metabolically inert but is a complex organ that can become active in the obese, insulin-resistant state leading to the production of various pro-inflammatory cytokines [54,55]. These cytokines include interleukin-1β (IL-1β), interleukin-6 (IL-6), interleukin-8 (IL-8), interleukin-18 (IL-18), complement component 3 (C3), TNF-α, PAI-1, adiponectin, leptin, resistin, apelin, vaspin and visfatin. There is evidence that these inflammatory mediators could play a role in the progression of liver disease from “simple” steatosis to NASH [56,57], and also that they may stimulate the differentiation of stellate cells in the liver into myofibroblast-like cells resulting in a more fibrogenic environment [58]. IL-1β, IL-6 and TNF-α are traditionally considered to be pro-inflammatory cytokines, and are all thought to play a role in the pathogenesis of NASH and its associated fibrosis [59,60]. More recently it has been suggested that the balance of pro- and anti-inflammatory mediators can lead to alterations in the gut microbiota and that this may have a significant impact on the progression of hepatic steatosis to NASH [61]. It has also been suggested that apoptosis of hepatocytes could be an important factor in liver damage and specifically progression to NASH [62,63]. Recent findings indicate that patients with a higher degree of insulin resistance exhibit greater evidence for apoptosis of hepatocytes in liver biopsy specimens of morbidly obese individuals, and it has been speculated that this may be mediated by inflammatory cytokines [64]. These studies all provide evidence for a causative link between insulin resistance and hepatic damage mediated in part by inflamed, endocrinologically-active adipose tissue.

There is also clinical evidence that insulin resistance and the metabolic syndrome can cause a worsening of liver disease. A retrospective study of 103 individuals with NAFLD examined histological findings from paired liver biopsy specimens with an average interval of 3 years [65]. The authors observed marked variability in the progression of histological features of NAFLD between the 2 time points, but noted that those individuals with diabetes were at higher risk than non-diabetic people for progression of fibrosis. It is also established in the literature that metabolic syndrome and type 2 diabetes are strongly associated with severe liver disease such as cirrhosis and hepatocellular carcinoma [66,67,68,69]. It appears from the literature that individuals with type 2 diabetes and NAFLD combined are at markedly greater risk of more severe liver disease than those with NAFLD alone, and their liver-related mortality is greater.

**Figure 1:**
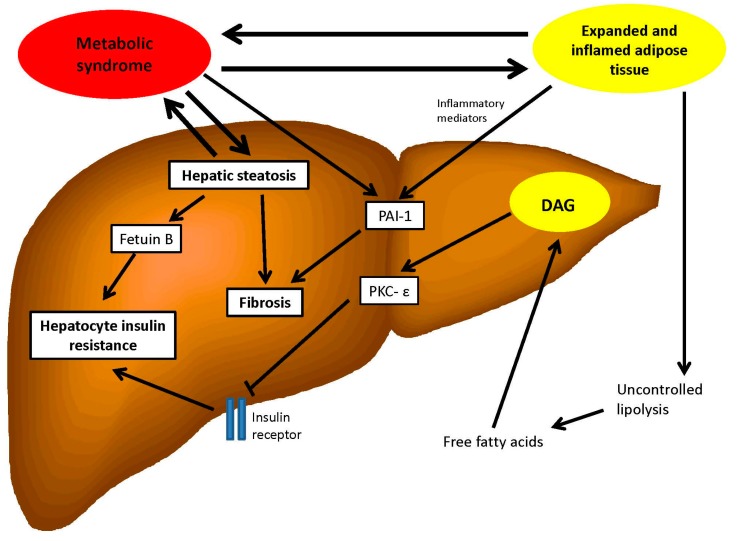
Schematic demonstrating the bidirectional interactions between hepatic steatosis and metabolic syndrome and aspects of how these are mediated. DAG: diacylglycerols; PKC-ε: protein kinase C-ε; PAI-1: plasminogen activator inhibitor-1.

There are a variety of cross-sectional studies available that demonstrate that metabolic syndrome and its components are associated with an increased risk of NAFLD in a variety of populations including North American [70], Mexican [71], Taiwanese [72] and Japanese [26]. However, given the cross-sectional nature of these studies they do not provide real evidence of a causative link. Of interest is a recent longitudinal prospective cohort study of 15,791 Han Chinese individuals followed up over a 6 years period [73]. They observed 3913 new cases of NAFLD in this population, and risk of incident NAFLD was markedly higher in those with metabolic syndrome. After adjusting for possible confounding factors such as age, diet, sex, smoking status and level of physical activity, the hazard ratio for incident NAFLD was found to be 1.94 (95% confidence intervals 1.78–2.13). The authors also observed that hazard ratios for incident NAFLD increased the more components of the metabolic syndrome were present at baseline, reaching 3.51 (95% confidence intervals 3.15–3.91) when 3 components were present as compared with individuals who exhibited no metabolic syndrome components. Figure 1 summarises the bidirectional relationship between hepatic steatosis and the metabolic syndrome with regards to the various aspects described above.

## 5. Evidence for a Disconnection between Hepatic Steatosis and Metabolic Syndrome

Despite the clear bidirectional causal links between NAFLD and the metabolic syndrome, there are certain situations where this appears to not be the case. In such scenarios there is a clinical disconnect between NAFLD and insulin resistance. Several groups have demonstrated that it is possible experimentally to induce either insulin resistance or hepatic steatosis individually without the presence of the other. The first evidence that hepatic steatosis could occur independently of insulin resistance was published in 2007 [74]. Here mice were raised which over-expressed acyl-CoA:diacylglycerol acyltransferase 2 (DGAT 2), an enzyme which acts to catalyze the final step of hepatic triglyceride biosynthesis. These mice were observed to develop marked hepatic steatosis in the absence of any abnormalities in plasma glucose and insulin levels, glucose and insulin tolerance, or infusion rates during hyperinsulinaemic euglycaemic clamp experiments. A subsequent study investigated variability in the DGAT2 gene to see if this relationship could also be found in humans. The authors investigated 187 individuals from south Germany, and observed 2 single nucleotide polymorphisms (SNPs) in DGAT2 that were associated with smaller decreases in liver fat following an exercise programme than wild type genotype [75]. There were no observed changes in insulin sensitivity among the different genotypes and thus the authors concluded that DGAT2 may play a role in mediating a disconnection between insulin resistance and hepatic steatosis. Additionally, it has been observed that inhibiting secretion of very low density lipoprotein (VLDL) from the liver by a genetic modification or diet-induced choline deficiency in a mouse model results in accumulation of hepatic triglyceride without causing insulin resistance [76,77].

More recently, there has been much interest focused on the patatin-like phospholipase domain-containing protein-3 (PNPLA3) gene, which encodes for a protein called adiponutrin. The exact role of this adiponutrin is currently unclear, however it is recognised as being a membrane-associated protein expressed in hepatic and adipose tissue that possesses lipogenic and lipolytic activities. There is evidence to suggest that it is located in lipid droplets and may play a role in triglyceride hydrolysis [78]. PNPLA3 gene expression is upregulated following the post-prandial insulin spike, and downregulated following fasting. It was reported in 2008 that a particular allele in PNPLA3 (I148M or rs738409) was strongly associated with increased hepatic steatosis and hepatic inflammation, with individuals homozygous for I148M exhibiting twice the level of hepatic fat content than non-carriers [79]. Interestingly, it was also observed that I148M carrier frequency was highest in Hispanic populations who are thought to have highest susceptibility to NAFLD, and regression analysis demonstrated that the presence or absence of this PNPLA3 variant along with another (453I) accounted for 72% of the observed ethnic differences in levels of hepatic steatosis from the Dallas Heart Study. It was subsequently reported that the I148M variant has a marked effect on enzyme activity and results in a disruption to normal hydrolysis of triglycerides leading to impaired secretion of very low density lipoproteins (VLDL) [80,81]. Interestingly, it has subsequently been demonstrated that the association between the I148M variant and NAFLD in independent of insulin sensitivity as measured by hyperinsulinaemic euglycaemic clamp, as well as central obesity [82,83]. Therefore the PNPLA3 I148M variant provides an example of how hepatic steatosis can occur in humans independently of insulin resistance and the metabolic syndrome.

**Figure 2:**
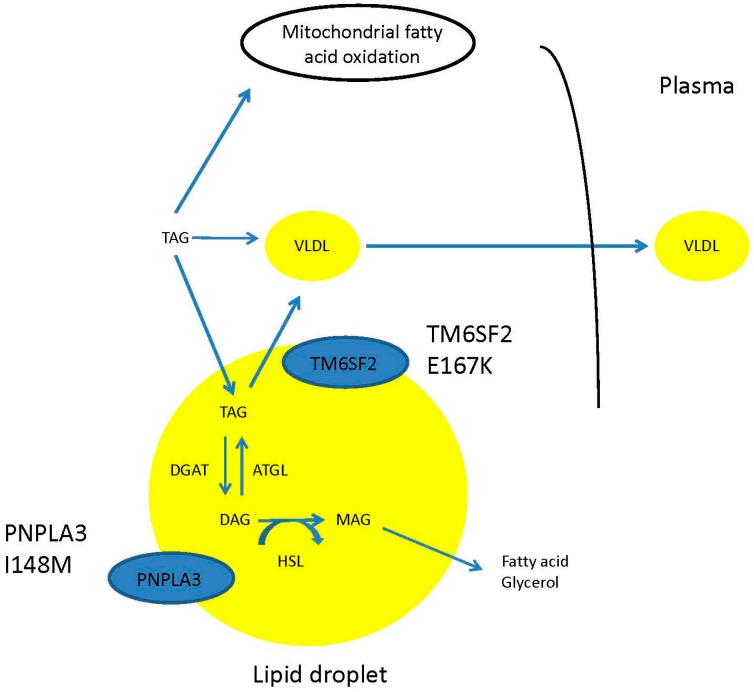
Interaction between PNPLA3 and TM6SF2 variants and lipid metabolism in the hepatic lipid droplet. TAG; triacylglycerol; DAG; diacylglycerol; MAG; monoacylglycerol; VLDL; very low density lipoprotein; DGAT; diglyceride acyltransferase; ATGL; adipose triglyceride lipase; HSL; hormone sensitive lipase.

A similar scenario has been identified more recently with the transmembrane 6 superfamily member 2 (TM6SF2) gene. TM6SF2 is expressed largely in the liver and intestine and is thought to play a key role in the regulation of hepatic fat metabolism and the secretion of triglyceride-rich lipoproteins. As with PNPLA3, it is thought to be located in lipid droplets and siRNA inhibition is associated with increased hepatocellular triglyceride concentration and lipid droplet lipid content [84]. Variation in this gene has been shown to be associated with susceptibility to NAFLD independently of variation in PNPLA3, with the variant being identified as E167K or rs58542926 [85]. The allele frequency of this variant was shown to be 7.2% in European populations. A subsequent study of 361 individuals, including 226 patients with biopsy-proven NAFLD, has shown that this variant has a modest effect on NAFLD susceptibility and is associated with a slightly higher risk of developing NASH [86]. A further study of 1074 individuals demonstrated an association between this variant and advanced fibrosis and cirrhosis that occurred independently of potential confounding factors such as age, BMI, presence of type 2 diabetes and PNPLA3 genotype status [87]. However, it should be noted that 2 studies looking at this variant in Japanese [88] and Chinese [89] populations of individuals with biopsy-proven NAFLD failed to show an association between it and fibrosis stage or general histological severity. The Japanese study had relatively small numbers with 211 individuals and just 2 who were homozygous for E167K, and it should be noted that both of these studies focused on a single ethnic group that may not be directly applicable to other populations. A meta-analysis of 10 published studies looked at the relationship between the E167K variant and the presence of NAFLD in a total of 5537 study participants [90]. This revealed a carrier frequency of up to 7%, and demonstrated a moderate effect on the risk of developing NAFLD with an odds ratio of 2.13 (95% confidence interval 1.36–3.30). Crucially, it has been shown in a recent Finnish study that this variant is associated with preserved insulin sensitivity and a lack of hypertriglyceridaemia suggesting that this represents a distinct subtype of NAFLD similar to that associated with the PNPLA3 I148M variant [91]. Figure 2 demonstrates the relationship between the 2 described genetic variants and the lipid droplet within the hepatocyte.

Further evidence for a dissociation between hepatic steatosis and insulin resistance may be found in the case of familial hypobetalipoproteinaemia (FHBL). Patients with FHBL have very low or absent levels of apolipoprotein B and this leads to an impairment of very low density lipoprotein export from the liver and consequently intra-hepatic accumulation of triglyceride. Amaro *et al.* [92] investigated a small number of overweight or obese patients with FHBL and observed that these individuals had greater insulin sensitivity than BMI- and hepatic triglyceride content-matched subjects with NAFLD alone. The authors speculate that this would support the assertion that hepatic steatosis is a marker rather than a cause of the metabolic syndrome, however this was a very small study and it is not clear how applicable these findings are to the wider population of people with NAFLD. It has also been observed that lysosomal acid lipase deficiency (LAL-D), a rare autosomal recessive inherited condition, can lead to hepatic steatosis in the absence of metabolic syndrome [93].

There is also evidence that adipose triacylglycerol lipase (ATGL) may play a role in a potential dissociation between insulin resistance and hepatic steatosis [94]. ATGL acts to initiate hydrolysis of stored lipid by selectively cleaving triacylglycerols and not diacylglycerols or monoacylglycerols. Knock-out studies have demonstrated that ATGL-deficient mice experience a marked hepatic steatosis [95] and similarly overexpression of the ATGL gene leads to a reduction in liver fat in mice [96]. One study investigated the effects of ATGL gene manipulation on insulin sensitivity in mice, and here the authors observed that while ATGL knock-out mice do develop marked hepatic steatosis this does not result in any changes to their hepatocyte insulin sensitivity [97]. Hepatic ATGL overproduction in the same mice resulted in reduced hepatic steatosis, and interestingly the authors did observe a mild increase in insulin sensitivity although this was not sufficiently large to result in improvements in fasting glucose concentrations or insulinaemia.

Further insights into a possible disconnection between hepatic steatosis and insulin resistance can be gained by looking at disorders of fatty acid oxidation. In health, fasting stimulates gluconeogenesis in the liver fuelled by oxidation of fatty acids. If fatty acid oxidation is impaired this can lead to fasting hypoglycaemia and accumulation of lipids resulting in hepatic steatosis [98]. In such situations individuals will exhibit enhanced glucose tolerance, therefore exhibiting the disconnection. This occurs in numerous inborn errors of fatty acid oxidation such as medium chain acyl-CoA dehydrogenase deficiency (MCADD) and carnitine palmitoyl transferase II (CPT-2) deficiency [99]. Additionally, peroxisome proliferator-activated receptor alpha (PPARα) stimulates the expression of many genes involved in fatty acid oxidation. Experimental mice who have undergone PPARα knock-out develop marked hepatic steatosis after being exposed to a high fat diet, and after fasting demonstrate hypoglycaemia and increased insulin sensitivity [100].

## 6. Conclusions

It is clear from the literature that there is a complicated causal relationship between NAFLD and the metabolic syndrome. NAFLD is considered by many to represent the hepatic manifestation of the metabolic syndrome however rigidly sticking to this dogma does not appreciate the complexity of the relationship. Clearly the two clinical entities share many aspects of their pathophysiology, and insulin resistance is at the centre of both. There is sufficient evidence now for not only reciprocal causality between these disease states, but also each acting as a perpetuating or exacerbating factor for the other.

There are, however, many aspects of the interactions between NAFLD and the metabolic syndrome that are yet to be fully elucidated, and this is clearly demonstrated by the situations where there is an apparent disconnect or dissociation between them. Arguably, the hepatic steatosis that occurs in these situations due to genetic variation and inborn errors of metabolic can be considered a separate clinical entity to that which is associated with insulin resistance and the metabolic syndrome. However, focusing on the mechanisms that underlie these observations of dissociation could prove valuable for identifying new therapeutic targets in metabolic disease.

## Figures and Tables

**Table 1:** Diagnostic criteria available for metabolic syndrome.

Criteria	WHO (1999)	NCEP (2001)	IDF (2005)	IDF (2009)
Required	Insulin resistance	Nil	Waist circumference ≥94 cm in men, ≥80 cm in women	Nil
Number of features	≥2 of:	≥3 of:	≥2 of:	≥3 of:
Obesity	Waist/hip ratio of >0.9 in men, >0.85 in women or BMI ≥ 30	Waist circumference ≥ 102 cm in men, ≥88 cm in women		Waist circumference—population specific definitions
Triglycerides	≥150 mg/dL (1.7 mmol/L)	≥150 mg/dL (1.7 mmol/L)	≥150 mg/dL (1.7 mmol/L)	≥150 mg/dL (1.7 mmol/L)
HDL-cholesterol	<40 mg/dL (1 mmol/L) in men, <50 mg/dL (1.3 mmol/L) in women	<40 mg/dL (1 mmol/L) in men, <50 mg/dL (1.3 mmol/L) in women	<40 mg/dL (1 mmol/L) in men, <50 mg/dL (1.3 mmol/L) in women	<40 mg/dL (1 mmol/L) in men, <50 mg/dL (1.3 mmol/L) in women
Hypertension	≥140/90 mmHg	≥135/85 mmHg	≥135/85 mmHg	≥135/85 mmHg
Glucose		110 mg/dL (6.1 mmol/L)	100 mg/dL (5.6 mmol/L)	100 mg/dL (5.6 mmol/L)
Microalbuminuria	Albumin/creatinine ratio > 30 mg/g; albumin excretion rate > 20 mcg/min			

WHO, World Health Organisation; NCEP, National Cholesterol Education Program; IDF, international diabetes federation.

**Table 2:** Characteristics of prospective studies linking hepatic steatosis to the development of type 2 diabetes.

Study	Country/Population	Sample Size	NAFLD Diagnostic Method/Surrogate Marker Used	Duration of Follow-Up	Key Findings	Limitations of Study
Vozarova 2002 [38]	Pima Indians aged 18–50	173 women, 278 men	ALT, AST and GGT concentrations	6.9 years average	High baseline ALT associated with increased risk of incident DM	Only surrogate markers used, no control for alcohol/hep C
Lee 2003 [39]	Korean men aged 25–55	4088 men	GGT concentration	4 years	Strong relationship between baseline GGT and risk of incident DM	Only studied men, only used surrogate marker
Hanley 2005 [40]	USA non-Hispanic whites and African American adults	910 women, 715 men	ALT, AST and ALP concentrations	5.2 years average	ALT and ALP in upper quartile at baseline significantly increased risk of metabolic syndrome	Only surrogate markers used for NAFLD diagnosis
Ekstedt 2006 [41]	Swedish NAFLD patients	87 men, 42 women	Biopsy-proven NAFLD	13.7 years average	Marked increase in proportion of patients with DM over period of study	No control group, no baseline glycaemic data to compare
Monami 2008 [42]	Florence aged 40–75	3124 total	ALT, AST and GGT concentrations	40 months average	Baseline GGT near upper limit of normal predicts incident DM	Study population participated in screening programme for diabetes, may not be representative
Goessling 2008 [43]	New England adults, all white	1575 women, 1237 men	ALT and AST concentrations	20 years	Increased ALT associated with higher risk of DM and metabolic syndrome, increased AST associated with incident DM risk	Homogenous study population, only surrogate markers used
Adams 2009 [29]	Western Australian adults	115 women, 243 men	NAFLD diagnosed with ALT after exclusion of other causes	11 years	NAFLD associated with higher risk of incident diabetes	Not an independent predictor if adjusted for WC, hypertension or insulin resistance
Ryu 2010 [44]	Korean men aged 30–65	9148 men	GGT concentrations	4.1 years average	Increase in GGT during study period predicted incident metabolic syndrome	Did not use accepted criteria for diagnosis of metabolic syndrome
Balkau 2010 [45]	Western France, aged 30–65	1950 women, 1861 men	NAFLD diagnosed using fatty liver index (FLI) score	9 years	Higher FLI score at baseline predicted incident DM	Used FLI rather than formal diagnostic methods
Sung 2011 [46]	Korean adults	7236 men, 3855 women	NAFLD diagnosed with ultrasound scan	5 years	Presence of fatty liver on ultrasound strongly predicted incident DM	Ultrasound relatively insensitive for diagnosis

ALT, alanine aminotransferase; AST, aspartate aminotransferase; GGT, gamma-glutamyl transferase; DM, diabetes mellitus; ALP, alkaline Phosphatase; NAFLD, non-alcoholic fatty liver disease; WC, waist circumference.

## Abbreviations

The following abbreviations are used in this manuscript:

NAFLD	Non-alcoholic fatty liver disease
PNPLA3	Patatin-like phospholipase domain-containing protein-3
TM6SF2	Transmembrane 6 superfamily member 2 protein
HDL-C	High density lipoprotein cholesterol
NASH	Non-alcoholic steatohepatitis
PKC-ε	Protein kinase C-ε
FLI	Fatty liver index
PAI-1	Plasminogen activator inhibitor-1
TNF-α	Tumour necrosis factor-α
IL	Interleukin
DAG	Diacylglycerols
DGAT	Diacylglycerol acyltransferase
SNP	Single nucleotide polymorphism
VLDL	Very low density lipoprotein
TAG	Triacylglycerol
MAG	Monoacylglycerol
ATGL	Adipose triglyceride lipase
HSL	Hormone sensitive lipase
FHBL	Familial hypobetalipoproteinaemia
BMI	Body mass index
